# Development an extended-information success system model (ISSM) based on nurses’ point of view for hospital EHRs: a combined framework and questionnaire

**DOI:** 10.1186/s12911-022-01800-1

**Published:** 2022-03-22

**Authors:** Zahra Ebnehoseini, Hamed Tabesh, Amir Deghatipour, Mahmood Tara

**Affiliations:** 1 Psychiatry and Behavioral Sciences Research Center, Mashhad University of Medical Sciences, Mashhad, Iran; 2 Department of Medical Informatics, Faculty of Medicine, Mashhad University of Medical Sciences, Mashhad, Iran; 3 Ibn-Sina Hospital, Mashhad University of Medical Sciences, Mashhad, Iran

**Keywords:** Hospital information system, Electronic health record, Evaluation, Information system success model

## Abstract

**Background:**

Understanding the hospital EHR success rate has great benefits for hospitals. The present study aimed to 1-Propose an extended-ISSM framework and a questionnaire in a systematic manner for EHR evaluation based on nurses’ perspectives, 2-Determine the EHR success rate, and 3-Explore the effective factors contributing to EHR success.

**Methods:**

The proposed framework was developed using ISSM, TAM3, TTF, HOT-FIT, and literature review in seven steps. A self-administrated structured 65-items questionnaire was developed with CVI: 90.27% and CVR: 94.34%. Construct validity was conducted using EFA and CFA. Eleven factors were identified, collectively accounting for 71.4% of the total variance. In the EFA step, 15 questions and two questions in EFA were excluded. Finally, 48 items remained in the framework including dimensions of technology, human, organization, ease of use, usefulness, and net benefits. The overall Cronbach’s alpha value was 93.4%. In addition, the hospital EHR success rate was determined and categorized. In addition, effective factors on EHR success were explored.

**Results:**

In total, 86 nurses participated in the study. On average, the “total hospital EHR success rate” was moderate. The total EHR success rates was ranging from 47.09 to 74.96%. The results of the Kruskal–Wallis test showed that there was a significant relationship between “gender” and “self-efficacy” (*p*-value: 0.042). A reverse relation between “years of experience using computers” and “training” (*p*-value: 0.012) was observed. “Years of experience using EHR” as well as “education level” (*p*-value: 0.001) and “ease of use” had a reverse relationship (*p*-value: 0.034).

**Conclusions:**

Our findings underscore the EHR success based on nurses’ viewpoint in a developing country. Our results provide an instrument for comparison of EHR success rates in various hospitals.

**Supplementary Information:**

The online version contains supplementary material available at 10.1186/s12911-022-01800-1.

## Background

Many studies in the literature support the idea that the adoption of Health Information Technology (HIT), and specifically the Electronic Health Record (EHR) in hospitals, provides great potential value to health care organizations [1]. Among these organizations, hospitals have complex environments that face a great deal of uncertainty such as information asymmetry [2]. In the past two decades, the e-health including EHR has become more popular among hospitals in Iran. SEPAS is the national hospital-based EHR for all Iranian citizens. The aim of this system is to connect hospitals and medical centers and to aggregate medical information at all levels of the healthcare system. All hospitals in Iran should adopt an EHR system and send inpatient and outpatient data to SEPAS [3]. Howerver, hospital EHRs have particular complexities [4]. Many studies suggest that EHR implementation alone is insufficient to effectively improve healthcare quality [5]. As such, various factors contribute to the successful implementation and use of EHR [6]. For example, human factors such as resistance to change [1, 7], low perceived uselessness [1], time and resource constraints, suboptimal clinic workflows, information access limitations, and insufficient clinician training [8] were the most frequent barriers regarding adoption of EHR in several studies. For the EHR systems to be successfully implemented and their potential impacts realized, it is essential that human and organizational processes are understood by users and involved in motivating change and adoption [9]. The interactive dimensions of the context, content, and process can shape organizational changes in hospitals and achieve the success of EHR implementation in hospitals depending on the effective interaction of the aforementioned dimensions [4]. The findings of the study by Ojo and Popoola revealed a highly significant relationship between technical, social, organizational, financial, and political factors and EHR success in hospitals [10]. These results were supported by the results of the Ciccomoello et.al study. Users and technology can collectively form the system and influence its acceptance and adoption. They believed that the expressed needs, the involvement of different users, and assessing system impacts on user's point of view were key factors affecting EHR adoption [9]. Moreover, the results of the study by Handayani et al. showed that non-technological dimensions, such as human and organizational dimensions, were more effective on EHR success than technological dimensions [11]. Therefore, user characteristics and business environment are important factors in adoption of EHRs.

Nurses are the largest user group of EHRs and provide health services in every clinical environment including hospitals [12]. EHR has great advantages for nurses in hospitals such as avoiding missing data, making nurses aware of the importance of documentation as a data source, adopting the use of evidence-based tools, avoiding customization, and seeking ways to use nursing documentation for research and quality measurement [13]. Rudin et al. suggested four EHR benefits for nurses including better clinical decisions, better triage decisions, better collaboration, and automation of tasks [14]. However, despite the expected benefits of EHR systems, the adoption of these system is different among nurses [15]. Krick and Tobias believed that the reasons for the lack or low acceptance of EHRs by nurses can be due to low usability of the system, no visible benefits for real work practice, as well as privacy issues. Scientific evaluations that offer the implementation status of the systems from different users' perspectives could help to understand the bigger picture of digital nursing technologies' success and provide important insights on specific impact factors. Comprehensive evaluation frameworks clearly show vital aspects of evaluation and play a significant role in supporting researchers, decision-makers, and developers. Evaluation frameworks can be used to provide a structure for the evaluation of nursing systems as well as information and definitions of technology success, evaluation areas, methods, and tools. Accordingly, an evaluation framework can facilitate a systematic approach in nursing systems evaluation [16]. In addition, the results of a literature review suggested that a modification of the existing frameworks may provide a better explanation of nurses’ acceptance and success of EHRs [12]. Hence, developing an EHR evaluation framework and understanding of the hospital EHR success rate based on nurses’ points of view has great benefits for hospitals about EHR adoption. The first objective of this research was to propose an extended-ISSM framework and a questionnaire in a systematic manner for EHR evaluation based on nurses’ perspectives. The second purpose was to determine the EHR success rate. The final goal was to explore the effective factors on EHR success in a hospital as a case study.

## Methods

### Research framework

**Fig. 1:**
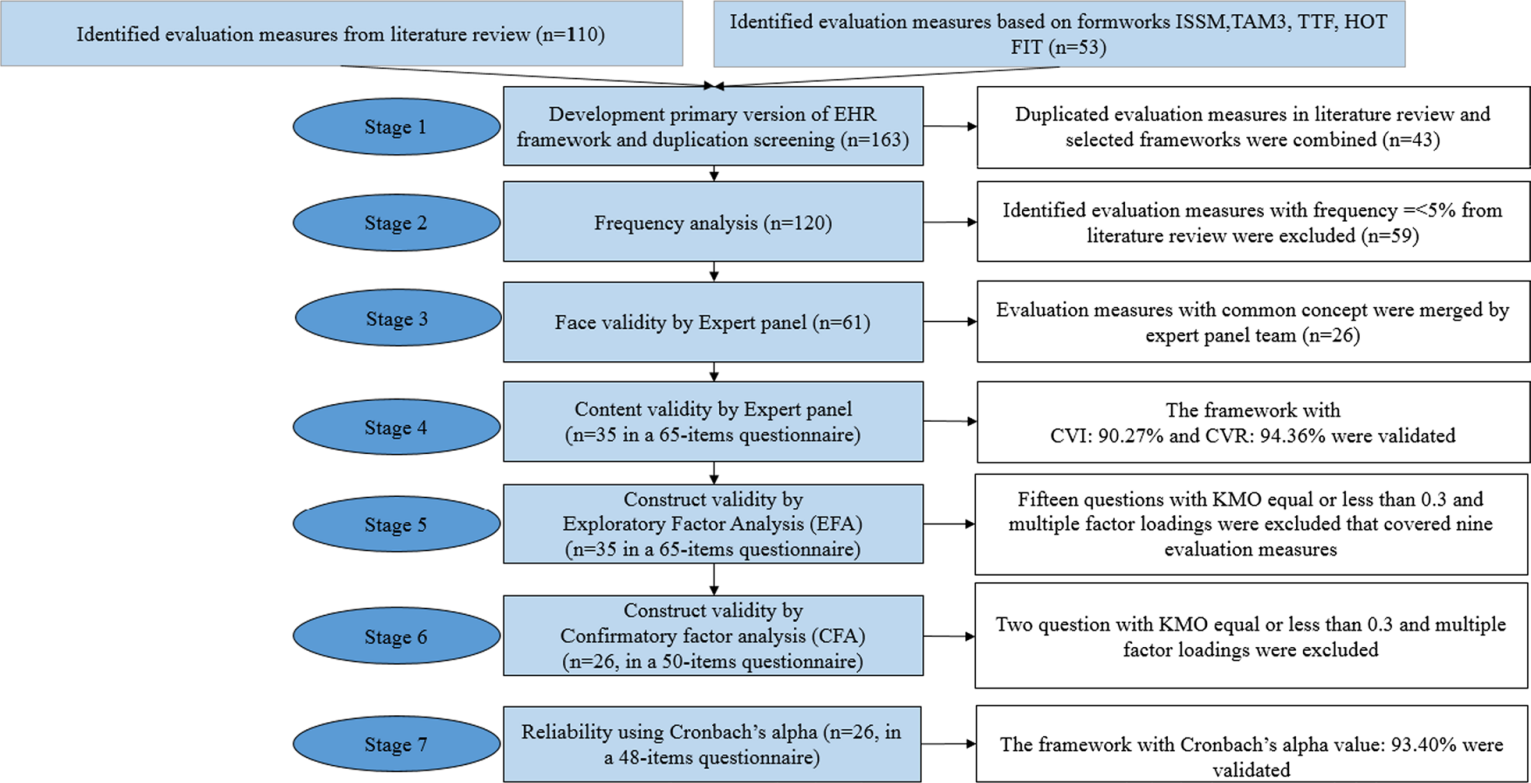
Summary of the research framework development steps

The research framework was developed in the following seven steps (Fig. 1).

#### Step 1: Development primary version of EHR framework and duplication screening

The proposed framework was developed mainly based on the Information Systems Success Model (ISSM) [17], Technology Acceptance Model (TAM2) [18], Task Technology Fit (TTF) [19], Human Organization Technology-Fit (HOT-FIT) [20], and our published literature review [21].

The ISSM is a well-validated evaluation framework and has seven dimensions. ISSM was developed by DeLone and Mclean [22] and was subsequently updated [17]. ISSM encompassed seven dimensions: system quality, information quality, service quality, system use, intention to use, satisfaction, and net benefits. System success depends on the interaction between its dimensions [17]. TAM was developed by Davis et al. in 1989 to predict users’ adaptation and use of new technology. It proved that individuals’ behavioral intention to use new technology is determined by two beliefs: perceived usefulness and perceived ease of use [23]. Venkatesh and Davis [18] proposed an extension of TAM known as TAM2 . The dimensions of TAM3 were as follows: perceived usefulness, perceived ease of use, computer self-efficacy, perceptions of external control computer, playfulness, computer anxiety, perceived enjoyment, subjective norm, voluntariness, image, job relevance, output quality, result demonstrability, behavioral intention, and use. Goodhue and Thompson suggested TTF in 1995. They believed that consistency between technology and tasks can increase users’ performance. TTF encompasses four dimensions including task characteristics, technology characteristics, task-technology fit, and performance/utilization [19]. HOT-FIT was developed by Yusof et al. in 2008 based on ISSM and IT-Organization Fit Model [24]. Our literature review was conducted to identify EHR evaluation frameworks that identified 110 evaluation measures to assess EHR from 62 eligible studies. In addition, 53 evaluation measures were identified from the selected frameworks including ISSM, TAM 3, TTF, and HOT-FIT. In the first step, the total identified evaluation measures (n = 163) were saved in an excel file. In this step, duplicate evaluation measures in each dimension were identified (n = 43). Subsequently, 120 evaluation measures were retrieved in six scopes in the following dimensions. Additional file 1 (the list, relative frequency, and the final status of the evaluation measures) shows the list, relative frequency, and the final status of each of the evaluation measures. The categorization of dimensions was based on our literature review.

- *Technology*: System quality, information quality, and service quality.
- *Human*: Satisfaction, system use, computer knowledge and self-efficacy, users’ characteristics and personality, and positive or negative feeling about EHR.
- *Organization*: Compatibility and fitness with the work process, social factors, management support, task equivocality, job relevance, image, environment, physician involvement, physician autonomy, communication, organization structure, coherence, cognitive participation, collective action, reflexive monitoring, monitoring and feedback, leadership, physical proximity, competition, employee understanding and support of implementation, organizational support for implementation, innovative culture in hospital, open culture in hospital, situational normality, strategy, supporting best practices, supportive norms, caseload, and voluntary turnover.
- Ease of use
- Usefulness
- *Net benefits*: Effects on outcome quality of care, effects on workflow and organization, and effects on the environment.

#### Step 2: Frequency analysis

Some evaluation measures were extracted from only one, two, or three studies. It is not possible for all evaluation measures to be included in an evaluation framework, because it can lead to low integrity and practicality of the framework. Hence, in the second step, frequency analysis was conducted. The evaluation measures with frequency =  < 5% (n <  = 3) from the literature review were excluded from the framework (n = 59).

#### Step 3: Face validity

Since the evaluation measures were collected from various resources, an expert panel team assessed the 61 remaining evaluation measures to merge them with the common concept. In the third step, 26 evaluation measures were merged. For example, the evaluation measure of "accessibility” in the dimension of "system quality" was merged with “sufficient resources”. The evaluation measures including “usability” and “confusion” in system quality dimension were merged with dimensions of “ease of use”. The list of merged evaluation measures are shown in Additional file 1.

#### Step 4: Content validity

In the fourth step, a self-administrated structured 65-items questionnaire that covered the 35 remaining evaluation measures were developed as follows (Additional file 2: Extended ISSM with a 65-items questionnaire for hospital EHRs based on nurses’ point of view validated by expert panel before Confirmatory Factor Analysis (CFA)):

- Technology (number of questions = 24):
  - System quality: sufficient resources, reliability, availability, system interoperability and integration with other information systems, response time.
  - Information quality: privacy and security, up-to-date, sufficiently, format, locatability, accuracy right level of detail, authorization, and timeliness.
  - Service quality: empathy, responsiveness, assurance, responsiveness and training.
- Organization (number of questions = 9): Management support, social support, and Task Technology Fit (TTF) and environment.
- Human (number of questions = 14): Self-efficacy, positive or negative feeling about EHR (including computer anxiety, result demonstrability, perceived enjoyment), users’ satisfaction, system use, voluntariness, image, and job relevance.
- Ease of use (number of questions = 4)
- Usefulness (number of questions = 4)
- Net benefits (number of questions = 10): effects on outcome quality of care and effects on workflow and organization.

Eleven experts participated in the expert panels including: three nurses, three medical informatics specialists, two health information management specialists, and three hospital HIS managers. Each expert had a long experience in the use of hospital EHR. Content validity ratio (CVR) and Content Validity Ratio (CVR) were calculated for each item by an expert panel. A CVR of zero or greater indicates that at least half of the experts deemed the item as “Essential” for the construct assessment [25]. An expert panel validated the questionnaire with CVI: 90.27% and CVR: 94.36%. No evaluation measure was excluded in this step.

#### Step 5: Construct validity with exploratory factor analysis (EFA)

In the fifth step, the validated 65-items questionnaire by the expert panel was used to construct validity, which was carried out using Exploratory Factor Analysis (EFA). A 5-point Likert scale ranging from “completely disagree” to “completely satisfy” was mainly used.

Data gathering was conducted in the largest psychiatric hospital and education center in eastern Iran. The hospital EHR has implemented in the case hospital ten years ago. Given the fact that human participants were involved in the current study, all methods were conducted based on the ethical standards of the Ethical Committee of Mashhad University of Medical Sciences. Scale development in factor analysis (EFA, CFA) are large sample size method because sample size affects precision and replicability of the results [26]. Therefore, all registered nurses in the EHR database who worked in the case hospital were invited to participate in the study (n = 112). The purpose of the study was explained and participants were assured that their confidentiality would be maintained. Participation in this study was voluntary, and the participants could withdraw from the study at any time. Questionnaires were provided to users who agreed to participate in this study. The written consent was obtained from all participants. The EFA was performed using Principal Component Analysis (PCA). The Kaiser–Meyer–Olkin (KMO) was conducted to measure sampling adequacy, and Bartlett’s Test of Sphericity was applied to assess whether factor analysis is appropriate. Varimax rotation was performed to identify the uncorrelated factors. The factor's extraction was consistent with the eigenvalue > 1 rule. Since only items with a loading factor ≥ 0.3 are acceptable for a specific factor, the threshold of factor loading was considered as 0.3 or greater [27, 28]. In this step, fifteen questions with KMO equal to or less than 0.3 and multiple factor loadings were excluded that covered nine evaluation measures, and 50 questions that belonged to 26 evaluation measures remained in the framework and were categorized based on results of EFA. Three evaluation measures of system quality including “reliability”, “availability”, and “system interoperability and integration with other information systems”, as well as two evaluation measures of authorization, and timeliness in information quality dimension were excluded from the framework. “Training” with two questions created a factor and was separated from “service quality”.

The evaluation measures including “system use”, “voluntariness”, “image”, and “job relevance” were categorized with “performance expectancy” named usefulness dimension. In addition, evaluation measures of “positive or negative feeling about EHR" dimension including computer anxiety, result demonstrability, and perceived enjoyment as well as users’ satisfaction were excluded from the human scope. One question in dimension of ease of use had multiple factor loading and was excluded.

The evaluation measure of ‘environment” in organization dimension was excluded from the framework and evaluation measure of “task equivocality”, “task interdependence”, and “compatibility and fitness with the work process” were grouped together and created the factor of TTF. Three evaluation measures encompass “effects on outcome quality of care”, “effects on workflow and organization”, and “privacy and security” were grouped as a factor that was named “net benefit”.

#### Step 6: Construct validity with confirmatory factor analysis (CFA)

In the sixth step, after conducting the EFA and excluding the questions with KMO equal or less than 0.3 and multiple factors, a Confirmatory Factor Analysis (CFA) was performed on the entire set of remaining items simultaneously (26 evaluating measures and 50 questions) to confirm the framework. CFA represents a powerful statistical technique used to determine whether the number of factors and pattern of item-factor loadings is consistent with what would be expected by a priori theory [29]. The method of CFA was the same as EFA. The results of the CFA showed that eleven factors with eigenvalues greater than 1.00 were identified, jointly accounting for 71.4% of the total variance. The value of KMO was 0.774, indicating sampling adequacy for the factor analysis. Bartlett’s test of Sphericity was statistically significant (*p*-value < 0.000). Out of 50 questions, the factor loading of one question was lower than 0.3, which was not excluded from the questionnaire by the research team. Two questions in information quality and social support dimensions had multiple factor loading removed from the questionnaire. Finally, 48 items remained in the study. According to the results of CFA, eleven factors were identified in the extended-ISSM questionnaire. Four factors including computer resource, information quality, service quality, and net benefits completely confirmed the original ISSM dimensions. Seven dimensions including training, task technology fit, social support, top management support, self-efficacy, ease of use, and usefulness were identified and considered as influencing factors on EHR success. Dimension of the “satisfaction” that is one of the ISSM dimensions was excluded in EFA. The 48-item questionnaire included six scopes as follows (Additional file 3: Extended ISSM with a 50-items questionnaire for hospital EHRs based on nurses’ point of view validated by Exploratory Factor Analysis (EFA)):

- Technology
  - System quality: sufficient resources (factor 1, n = 3).
  - Information quality (factor 2, n = 7): up-to-date, sufficiently, format, locatability, accuracy, and right level of detail.
  - Service quality (factor 3, n = 5): empathy, responsiveness, assurance, responsiveness.
  - Training (factor 4, n = 2)
- Organization
  - Management support (factor 5, n = 2)
  - social support (factor 6, n = 2)
- Human: Self-efficacy (factor 8, n = 2).
- Ease of use (factor 9, n = 3)
- Usefulness (factor number 10, including evaluation measures of “performance expectancy”, “system use”, “voluntariness”, “image, and “job relevance”, n = 8).
- Net benefits (factor number 11 including evaluation measures of “effects on outcome quality of care”, “effects on workflow and organization”, and “privacy and security”, n = 11).

#### Step 7: Reliability of the framework

In the seventh Step, the reliability of the 48-item questionnaire was measured by Cronbach’s alpha. Table 1 shows the dimensions and questions of the proposed questionnaire. The overall Cronbach’s alpha value of the instrument was determined as 93.40%, demonstrating high reliability. The value of Cronbach’s alpha was very high among the six dimensions of computer resource, information quality, service quality, training, ease of use, usefulness, and net benefits. The range of values of Cronbach’s alpha in all dimensions was from 40 to 91.7%. The low value of Cronbach’s alpha belonged to the self-efficacy dimension, and its value was 40.0%. Table 1 and Additional file 3 show all items of the proposed framework including remaining and excluded items.

**Table 1:** The results of the CFA and reliability of the proposed framework and 50-items questionnaire

Scope	Evaluation dimensions	Evaluation measures (Cronbach’s alpha % for the remained evaluation measures)	Questions	Factor loadings of CFA
Technology	System quality	Sufficient resources (Cronbach’s α: 86.4)	CR1: Computer equipment (PC, monitor, keyword, and mouse)	0.86
			CR2: Intranet (local hospital network)	0.791
			CR3: Internet	0.783
	Information quality (Cronbach's α: 86.4)	Up-to-date	IQ1: The hospital EHR provide up-to-date information	0.738
		Sufficiently	IQ2: The hospital EHR covers your departments’ workflow and The hospital EHR	0.728
		Format	IQ3: Information field and reports in the hospital EHR appears orderly and easy to read	0.707
			IQ4: The hospital EHR s’ field labels and fields clearly and distinctively	0.533
		Locatability	IQ5: It is easy to find the information you need in hospital EHR	0.693
		Accuracy	IQ6: The hospital EHR’ data and information is compatible with paper medical record	0.662
		Right level of detail	IQ7: The hospital EHR provides sufficient and detailed information that seems to be just exactly what you need	0.621
		Authorization	IQ8: Privileges required to access the HIS restrict accessibility to necessary patient information for daily tasks	Multiple factor loading^a^
	Service quality (Cronbach's α: 85.6)	Empathy	SQ1: IT staff take your job problems seriously and interest to solve the problems	0.828
		Responsiveness	SQ2: IT staff provide their IT support services at the times they promise to do so	0.796
		Assurance	SQ3: You feel that IT staffs understand the health care objectives and they can communicate with you in familiar medical terms that are consistent	0.651
		Responsiveness	SQ4: The period time between a service request and IT staffs response is acceptable. (.e.g. solving a problem, giving authorized access to the hospital EHR components, and install new features)	0.633
			SQ5: You received the appropriate levels of training that you need to be able to understand and use the hospital EHR	0.595
		Training	T1: The hospital EHR Manual deliver a detailed user’s manual in printed and/or electronic form	0.83
			T2: The hospital EHR has a clear instruction manual that makes it easy for you to understand and operate	0.828
Human	Computer knowledge and Self-efficacy	Self-efficacy (Cronbach’s α: 40.0)	SE1: If there was no one around to tell you what to do as you go	0.645
			SE2: If you could call someone for help if you got stuck	0.534
Organization	Task Technology Fit (TFF) (Cronbach’s α: 58.9)	Task equivocality	TTF1: You frequently deal with business problems duo to ill-defined hospital EHR work flow	0.823
		Task interdependence	TTF2: The hospital EHR problem negatively effect on your performance	0.729
		Compatibility and fitness with the work process	TTF3: The hospital EHR s’ field are relevance to yours’ clinical and administrative workflow	0.383
	Social Support (SS) (Cronbach’s α: 68.8)	–	SS1: your colleagues who influence my behavior think that you should use the hospital EHR	0.576
			SS2: your colleagues in your department think that you should use the system	0.27
			SS3: The senior management of this business has been helpful in the use of the system	Multiple factor loading^a^
	Management support (Cronbach’s α: 65)	–	TM1: Senior management ask you opinion about hospital EHR improvement	0.755
			TM2: Top management making available sufficient resources for hospital EHR development	0.712
Usefulness (Cronbach’s α: 91.5)	Performance expectancy	–	UF1: Using the hospital EHR in your job increases your productivity	0.774
			UF2: Using the hospital EHR enhances the quality of the tasks you perform	0.758
			UF3: In your job, usage of the hospital EHR is important	0.697
			UF4: Using the hospital EHR in your job would enable you to do tasks more quickly	0.679
	System use	–	UF5: You want to use the hospital EHR	0.663
	Voluntariness	–	UF6: Your use of the system is voluntary	0.627
	Image	–	UF7: People in your hospital who use the hospital EHR have a high profile	0.653
	Job Relevance	–	UF8: You find hospital EHR to be useful in your job	0.452
Ease of Use (Cronbach’s α: 82.7)	–	–	EU1: Interacting with the hospital EHR does not require a lot of my mental effort	0.855
			EU2: You find it easy to get the hospital EHR to do what you want it to do	0.829
			EU3: It would be easy for you to become skillful at using the hospital EHR	0.759
Net benefit (Cronbach’s α: 91.7)	Effects on outcome quality of care	–	NB1: The hospital EHR S improves the quality of care	0.826
			NB2: By using the hospital EHR, patients have a better insight into the care provided by health care providers	0.749
			NB3: The hospital EHR reduces medical errors and improves patient safety	0.735
			NB4: The hospital EHR increases to health professionals’ ability to make patient care decisions	0.688
	Effects on work flow and organization	–	NB5: The hospital EHR decreases the wastefulness of resources and costs in the hospital	0.689
			NB6: The hospital EHR reduces patients waiting time for health care at the hospital	0.688
			NB7: The hospital EHR reduces the referral of patients or their families to different hospital departments	0.632
			NB8: The hospital EHR facilitates continuity of care in the next patient encounters	0.591
			NB9: The hospital EHR increases hospital administration ‘s control on patient cost	0.587
			NB10: Using the hospital EHR facilitates communication between various health professionals when patient is re-admitted, is referred to other organizations and is received follow-up outpatient care	0.49
	Privacy and security	–	NB11: The hospital EHR enhances the safety and confidentiality of patient data	0.645

The cells noted with a superscript “a” shows the excluded questions in CFA

### Statistics

Participants’ responses were tabulated and scores for invert statements were reversed. No statistical imputation was performed for missing data. Descriptive summary statistics were calculated as frequencies and percentages for demographics. Means rate and 95% Confidence Interval (CI) of hospital EHR success for the dimensions of extended-ISSM were calculated based on nurses’ perspectives.

Hospital EHR success rate in the case hospital was calculated in three steps. First, the rate of hospital EHR success based on nurses' point of view was determined for each of the evaluation measures and then total dimensions of the extended-ISSM questionnaire by all nurses. Finally, the mean of the hospital EHR success rate was categorized as follows: 1- Appropriate (75% ≤ hospital EHR success rate), 2- Moderate (50% ≤ hospital EHR success rate < 75%), 3- Low (25% ≤ hospital EHR success rate < 50%), and 4- Poor (coverage rate < 25%). For more details about the hospital EHR success rate measurement, see our previous papers [30, 31].

The data distribution of EHR success dimensions for different levels of effective factors on each dimension was determined using the Shapiro–Wilk test of normality and Kolmogorov–Smirnov. The dependent variables were dimensions of extended-ISSM and independent variables were age (30 > / 30–40/ 40 <), gender, educational level (bachelor/master), ICDL certification (yes/no), nursing status (nurses/ head nurses), number of shifts per day (1/2), years of work experience (< 5/ 6–10/ > 10), years of experience using computers (< 3/ ≥ 3), and years of experience using EHR (< 6/ ≥ 6). As in all dimensions of extended-ISSM were found to have a non-normal distribution, comparison tests were conducted using Mann–Whitney U tests for two-level variables and Kruskal–Wallis for three or more level variables. A *p*-value of less than 0.05 was considered statistically significant. Non-normally distributed data were reported as medians and Interquartile Ranges (IQR) in the current study. Data analysis was performed using SPSS, version 26 statistical software.

## Results

### Participants

A total of 112 questionnaires were distributed among all invited nurses. Finally, 86 valid questionnaires were collected (Response rate 76.7%). The participants were 68 nurses and 18 head nurses. Fifty-one of the participants were female, and 35 were male. Most of the cases (n = 49, 57.0%) were aged 31–40. Eighteen cases were 30 years old, and nineteen were over 40 years old. In addition, 80.2% of nurses had Bachelor’s degree and 18.8% had a Master’s degree.

Most of the participants (n = 35, 40.7%) had more than ten years of work experience. Moreover, 38.4% and 20.9% of the participants had 5–10 and less than 5 years of work experience, respectively. Fifty-five participants had one shift per day. The majority of the participants (n = 71, 82.6%) had more than three years of experience to work with computer, and also, had over six years of EHR experience (n = 65, 75.6%).

## Reliability and validity

### Mean rate of hospital EHR success

**Fig. 2:**
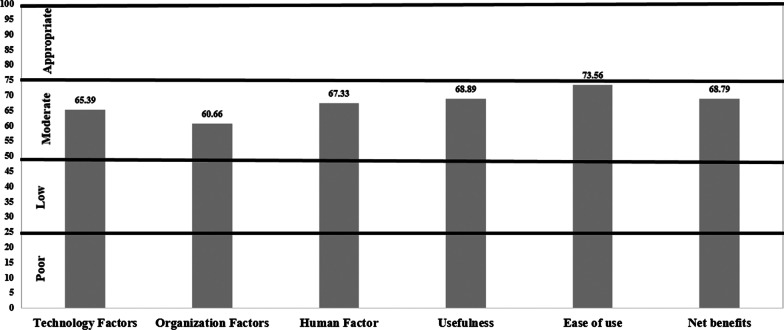
The Mean of hospital EHR success rate based on nurses’ point of view

On average, the “total hospital EHR success rate” was 66.81% (95% CI: 64.69%, 68.93%) that were categorized in the “moderate” group. The range of “total EHR success” rate was from 45.35% (95% CI 40.96, 49.73) to 74.96% (95% CI: 70.61, 79.30). The dimensions of “computer resource”, “ease of use” and “social support” acquired the highest hospital EHR success rate, respectively. In addition, the dimensions of “training” and “top management support” had the lowest rates of hospital EHR success rate. Low managerial support and training may lead to none of the dimensions being categorized in the "appropriate" category. It seems that due to the shortage of training courses, the users relied on self-efficacy and social support. As shown in Fig. 2, the overall dimension of "ease of use" acquired the highest success rate, followed by “usefulness” and “net benefit”. The case HIS was used in the hospital for more than 10 years. As such, most users realized the benefits and applications of the EHR in the workflow. Table 2 presents the mean success rates for all dimensions of hospital EHR evaluation based on the nurses’ point of view. Figure 2 shows the total hospital EHR success rate in departments of the case hospital.

**Table 2:** Mean of hospital EHR success rate based on extended-ISSM

Dimensions	Hospital EHR success categories*			
	Poor (Mean %, 95% CI)	Low (Mean %, 95% CI)	Moderate (Mean %, 95% CI)	Appropriate (Mean %, 95% CI)
*Technology factors*				
Computer resource^a^	–	–	74.96 (70.61, 79.30)	–
Information quality^a^	–	–	66.27 (63.75, 68.80)	–
Service quality^a^	–	–	66.41 (62.89, 69.94)	–
Training^b^	–	45.35 (40.96, 49.73)		–
*Organization factors*				
Task technology fit^b^	–	–	65.27 (62.54, 68.00)	
Social support^b^	–		71.16 (67.25, 75.07)	–
Top management support^b^	–	47.09 (43.11, 51.08)		–
*Human factors*				
Self-efficacy^b^	–	–	67.33 (62.48, 72.17)	–
Usefulness^b^	–	–	68.89 (65.52, 72.26)	–
Ease of use^b^	–	–	73.56 (70.53, 76.59)	–
Net benefits^a^	–	–	68.79 (65.64, 71.94)	–
Total Hospital EHR Success rate	–	–	66.81 (64.69, 68.93)	–

*(Hospital EHR success categories): Appropriate (75% ≤ hospital EHR success rate), Moderate (50% ≤ hospital EHR success rate < 75%), Low coverage (25% ≤ hospital EHR success rate < 50%), and Poor (coverage rate < 25%)

Cells noted with a superscript “a” shows the original dimensions of ISSM and superscript “b” refers to the added dimensions to the ISSM

### Effective factors on hospital EHR success

The results of the Mann–Whitney U tests and Kruskal–Wallis test showed that there was a significant statistical relationship between the variables of “gender” and “self-efficacy” (*p*-value: 0.042) (Table 3). The median “self-efficacy” in females (Median: 80%, Q1-Q3: 60%-90%) was higher than males (Median: 60%, Q1–Q3: 50–80%) (Table 4). There was not a significant statistical relationship between the variables of age, ICDL certification, and nursing status. Currently, as part of the employment process, new nurses are required to have ICDL certification. In addition, in recent years, computers and use of internet have become more common in Iran, particularly among the youth. All nurses and head nurses use EHRs as their daily routine in the case hospital. Hence, no significant relationship was observed between the variables of age, nursing status as well as ICDL certificate and different dimensions. Three significant relationships were observed as follows:

**Table 3:** Medians and interquartile ranges (IQR) in dimensions of proposed framework based on nurses’ characteristics

Participants’ characteristics	Items (frequency)	Technology				Organization			Human	Ease of use (EU)	Usefulness (UF)	Net benefits
		Computer resources (CR)	Information quality (IQ)	Service quality (SQ)	Training (T)	Task technology fit (TFF)	Social support (SS)	Top management support (TM)	Self-efficacy (SE)			
Age	30 > (18.00)	93.33 (66.67, 100.00)	71.43 (54.29, 77.14)	68.00 (52.00, 84.00)	40.00 (30.00, 60.00)	66.67 (53.33, 80.00)	70.00 (60.00, 90.00)	50.00 (30.00, 60.00)	60.00 (50.00, 90.00)	80.00 (66.67, 80.00)	72.50 (52.50, 80.00)	65.45 (50.91, 80.00)
	30–41 (49.00)	73.33 (56.67, 86.67)	62.86 (60.00, 74.29)	68.00 (56.00, 80.00)	40.00 (20.00, 60.00)	66.67 (60.00, 73.33)	70.00 (60.00, 80.00)	40.00 (40.00, 60.00)	70.00 (60.00, 90.00	66.67 (60.00, 80.00)	67.50 (61.25, 78.75)	70.91 (58.18, 80.00)
	40 < (19.00)	93.33 (66.67, 100.00)	71.43 (54.29, 77.14)	68.00 (52.00, 84.00)	40.00 (30.00, 60.00)	66.67 (53.33, 80.00)	70.00 (60.00, 90.00)	50.00 (30.00, 60.00)	60.00 (50.00, 90.00)	80.00 (66.67, 80.00)	72.50 (52.50, 80.00)	65.45 (50.91, 80.00)
Gender	Men (35.00)	86.67 (66.67, 100.00)	68.57 (60.00, 77.14)	68.00 (52.00, 80.00)	50.00 (30.00, 70.00)	66.67 (53.33, 66.67)	70.00 (60.00, 80.00)	40.00 (40.00, 60.00)	**60.00 (50.00, 80.00)a**	73.33 (66.67, 80.00)	67.50 (60.00, 80.00)	72.73 (60.00, 80.00)
	Female (51.00)	73.33 (53.33, 93.33)	65.71 (60.00, 74.29)	68.00 (56.00, 76.00)	40.00 (20.00, 60.00)	66.67 (60.00, 73.33)	70.00 (60.00, 80.00)	40.00 (30.00, 50.00)	**80.00 (60.00, 90.00)a**	80.00 (60.00, 80.00)	70.00 (60.00, 80.00)	65.45 (58.18, 78.18)
Education level	Bachelor (69.00)	73.33 (60.00, 93.33)	65.71 (60.00, 74.29)	68.00 (56.00, 80.00)	40.00 (30.00, 60.00)	66.67 (60.00, 73.33)	70.00 (60.00, 80.00)	40.00 (40.00, 60.00)	60.00 (60.00, 80.00)	**73.33 (60.00, 80.00)a**	70.00 (60.00, 80.00)	69.09 (59.09, 76.36)
	Master (17.00)	80.00 (60.00, 96.67)	65.71 (58.57, 75.71)	64.00 (50.00, 84.00)	50.00 (25.00, 60.00)	66.67 (60.00, 73.33)	70.00 (60.00, 90.00)	40.00 (30.00, 60.00)	60.00 (50.00, 90.00)	**80.00 (80.00, 100.00)a**	65.00 (60.00, 86.25)	70.91 (60.91, 85.45)
ICDL certification	No (12.00)	83.33 (65.00, 98.33)	68.57 (63.57, 74.29)	72.00 (54.00, 80.00)	50.00 (30.00, 60.00)	66.67 (50.00, 76.67)	70.00 (62.50, 90.00)	40.00 (40.00, 70.00)	65.00 (30.00, 87.50)	80.00 (70.00, 85.000)	72.50 (60.00, 81.88)	69.09 (63.64, 75.91)
	Yes (74.00)	73.33 (60.00, 93.33)	64.29 (59.29, 75.00)	66.00 (56.00, 77.00)	40.00 (27.50, 60.00)	66.67 (60.00, 73.33)	70.00 (60.00, 80.00)	40.00 (30.00, 60.00)	60.00 (60.00, 90.00)	73.33 (60.00, 80.00)	68.75 (60.00, 80.00)	70.00 (58.18, 78.64)
Nursing status	Nurse (68.00)	73.33 (60.00, 93.33)	65.71 (60.00, 74.29)	66.00 (56.00, 79.00)	40.00 (30.00, 60.00)	66.67 (60.00, 73.33)	70.00 (60.00, 80.00)	40.00 (30.00, 57.50)	60.00 (60.00, 80.00)	73.33 (60.00, 80.00)	68.75 (60.00, 80.00)	70.91 (60.00, 77.73)
	Head nurse (18.00)	86.67 (66.67, 100.00)	67.14 (58.57, 76.43)	74.00 (51.00, 88.00)	50.00 (20.00, 62.50)	66.67 (60.00, 73.33)	80.00 (67.50, 90.00)	50.00 (40.00, 60.00)	75.00 (60.00, 90.00)	80.00 (66.67, 81.67)	72.50 (61.88, 80.63)	64.55 (55.91, 82.27)
Number of shifts per day	1.00 (55.00)	73.33 (53.33, 93.33)	65.71 (60.00, 77.14)	68.00 (56.00, 80.00)	40.00 (20.00, 60.00)	66.67 (60.00, 73.33)	70.00 (60.00, 80.00)	40.00 (30.00, 50.00)	70.00 (60.00, 90.00)	80.00 (60.00, 80.00)	70.00 (60.00, 80.00)	69.09 (60.00, 78.18)
	2.00 (31.00)	80.00 (60.00, 93.33)	62.86 (60.00, 74.29)	64.00 (56.00, 80.00)	40.00 (30.00, 70.00)	66.67 (53.33, 66.67)	70.00 (60.00, 80.00)	40.00 (40.00, 60.00)	60.00 (60.00, 80.00)	73.33 (66.67, 80.00)	67.50)60.00, 80.00)	70.91 (60.00, 76.36)
Years of work experience (years)	< 5 (18.00)	60.00 (60.00, 95.00)	68.57 (62.86, 77.86)	74.00 (58.00, 81.00)	60.00 (37.50, 72.50)	60.00 (46.67, 70.00)	70.00 (60.00, 90.00)	40.00 (30.00, 72.50)	75.00 (50.00, 90.00)	80.00 (66.67, 86.67)	73.75 (80.63) 86.67	71.82 (63.18, 79.55)
	6–10 (33.00)	80.00 (56.67,90.00)	62.86 (57.14, 71.43)	64.00 (56.00, 76.00)	40.00 (20.00, 60.00)	66.67 (60.00, 73.33)	70.00 (60.00, 80.00)	40.00 (40.00, 50.00)	60.00 (60.00, 90.00)	73.33 (66.67, 80.00)	67.50(60.00, 77.50)	65.45 (57.27, 76.36)
	> 10 (35.00)	73.33 (60.00, 100.00)	65.71 (60.00, 77.14)	64.00 (52.00, 84.00)	40.00 (30.00, 60.00)	66.67 (60.00, 73.33)	70.00 (60.00, 80.00)	40.00 (30.00, 60.00)	60.00 (60.00, 80.00)	80.00 (60.00, 80.00)	67.50 (60.00, 80.00)	72.73 (54.55, 80.00)
Years of experience using computers (years)	≥ 3 (15.00)	80.00 (60.00, 93.33)	65.71 (62.86, 77.14)	76.00 (60.00, 84.00)	**60.00 (40.00, 70.00)**	66.67 (53.33, 73.33)	70.00 (70.00, 80.00)	40.00 (40.00, 70.00)	70.00 (60.00, 90.00)	66.67 (60.00, 80.00)	75.00 (65.00, 80.00)	63.64 (60.00, 76.36)
	< 3 (71.00)	73.33 (60.00, 93.33)	65.71 (60.00, 74.29)	64.00 (56.00, 76.00)	**40.00 (20.00, 60.00)**	66.67 (60.00, 73.33)	70.00 (60.00, 80.00)	40.00 (30.00, 50.00)	60.00 (60.00, 90.00)	73.33 (66.67, 80.00)	67.50 (60.00, 80.00)	70.91 (58.18, 78.18)
Years of experience using HIS (years)	< 6 (21.00)	80.00 (60.00, 90.00)	68.57 (61.43, 74.29)	64.00 (52.00, 80.00)	50.00 (30.00, 60.00)	60.00 (53.33, 70.00)	70.00 (65.00, 80.00)	40.00 (35.00, 65.00)	60.00 (50.00, 80.00)	**80.00 (70.00, 83.33)a**	70.00 (60.00, 80.00)	70.91 (61.82, 74.55)
	≥ 6 (65.00)	73.33 (60.00, 96.67)	65.71 (58.57, 75.71)	68.00 (56.00, 80.00)	40.00 (25.00, 60.00)	66.67 (60.00, 73.33)	70.00 (60.00, 80.00)	40.00 (35.00, 55.00)	60.00 (60.00, 90.00)	**73.33 (60.00, 80.00)a**	67.50 (60.00, 80.00)	69.09 (57.27, 80.00)

Medians and interquartile ranges (IQR) for the significant results in each dimension are bolded and indicated by letters a (*P* < 0.05)

**Table 4:** Effective factors on hospital EHR success in dimensions of proposed framework based on nurses’ characteristics

Participants’ characteristics	Non-parametric tests	Technology				Organization			Human	Ease of use (EU)	Usefulness (UF)	Net benefits
		Computer resources (CR)	Information quality (IQ)	Service quality (SQ)	Training (T)	Task technology fit (TFF)	Social support (SS)	Top management support (TM)	Self-efficacy (SE)			
Age	Kruskal–Wallis H	4.818	2.615	0.767	2.113	0.529	0.904	1.014	5.065	3.55	0.289	0.438
	Asymp. Sig	0.09	0.27	0.682	0.348	0.767	0.636	0.602	0.079	0.17	0.865	0.803
Gender	Mann–Whitney U	− 1.953	− 0.463	− 0.048	− 1.452	− 1.673	− 0.852	− 0.678	− 2.031	− 0.197	− 0.088	− 0.849
	Asymp. Sig. (2-tailed)	0.051	0.643	0.961	0.147	0.094	0.394	0.498	**0.042 **^**a**^	0.844	0.93	0.396
Education level	Mann–Whitney U	− 0.279	− 0.441	− 0.332	− 0.75	− 0.745	− 0.216	− 1.025	− 0.083	− 3.378	− 0.071	− 0.755
	Asymp. Sig. (2-tailed)	0.78	0.659	0.74	0.454	0.456	0.829	0.305	0.934	**0.001 **^**a**^	0.944	0.45
ICDL certification	Mann–Whitney U	− 1.051	− 1.113	− 0.544	− 0.374	− 0.254	− 0.426	− 0.089	− 0.345	− 1.55	− 0.331	− 0.343
	Asymp. Sig. (2-tailed)	0.293	0.266	0.587	0.709	0.8	0.67	0.929	0.73	0.121	0.741	0.731
Nursing status	Mann–Whitney U	− 1.474	− 0.676	− 0.628	− 0.432	− 0.432	− 1.397	− 0.759	− 1.338	− 1.277	− 1.006	− 0.287
	Asymp. Sig. (2-tailed)	0.141	0.499	0.53	0.666	0.666	0.162	0.448	0.181	0.202	0.315	0.774
Number of shifts per day	Mann–Whitney U	− 0.976	− 0.338	− 0.676	− 0.96	− 1.227	− 0.702	− 0.377	− 0.742	− 0.642	− 0.302	− 0.032
	Asymp. Sig. (2-tailed)	0.329	0.735	0.499	0.337	0.22	0.483	0.706	0.458	0.521	0.763	0.975
Years of work experience (years)	Kruskal–Wallis H	1.378	5.018	1.566	4.442	1.972	0.854	0.526	1.221	2.524	1.003	1.644
	Asymp. Sig	0.502	0.081	0.457	0.108	0.373	0.652	0.769	0.543	0.283	0.606	0.44
Years of experience using computers (years)	Mann–Whitney U	− 0.057	− 0.582	− 1.678	− 2.499	− 0.533	− 0.238	− 1.012	− 0.274	− 0.928	− 1.301	− 0.239
	Asymp. Sig. (2-tailed)	0.954	0.56	0.093	**0.012 **^**a**^	0.594	0.812	0.312	0.784	0.353	0.193	0.811
Years of experience using HIS (years)	Mann–Whitney U	− 0.539	− 0.848	− 0.808	− 1.075	− 0.287	− 0.287	− 2.122	− 0.431	− 0.203	− 0.519	− 0.096
	Asymp. Sig. (2-tailed)	0.839	0.603	0.59	0.396	0.283	0.774	0.666	0.419	**0.034 **^**a**^	0.774	0.924

The significant results in each dimension are bloded and indicated by letters a (*P* < 0.05)

A reverse relation between the variable of “years of experience using computers” and the dimension of “training” (*p*-value: 0.012) as well as “years of experience using EHR” and the dimension of “ease of use” was observed (*p*-value: 0.034).

The median of the “training” dimension in “less than three years of experience in the computer” group (Median: 60%, Q1–Q3: 40–70%) was higher than the other group (Median: 40%, Q1–Q3: 20–60%). The median of the “ease of use” dimension in group of “less than six years of experience” using the EHR (Median: 80%, Q1–Q3: 70–83.33%) was higher than the other group (Median: 73.33%, Q1–Q3: 60–80%).

There was a significant difference in the “ease of use” dimension and education level groups (*p*-value: 0.001). The median of the “ease of use” dimension in the Master group (Median: 80%, Q1–Q3: 80–100%) was higher than in the Bachler group (Median: 73.33%, Q1–Q3: 60–80%). According to our results, a significant statistical relation between other dimensions and the independent variables was not observed. There was a significant relationship between experience in the use of computers and HIS and ease of use as well as training. The user with more level of computer and EHR felt the need to continue with the training courses.

## Discussion

In this study, evaluation measures were extracted from a comprehensive literature review and were combined with the most famous evaluation frameworks for EHR. Using a systematic method, more frequent and applicable evaluation measures to assess EHR were identified and nurses participated in the face, content, and construct validity. Finally, an extended-ISSM based on nurses’ points of view for hospital EHRs was developed. In addition, the mean of success rate based on nurses' perspectives was determined. The proposed evaluation framework and a questionnaire as well as the proposed method of calculating the success rate can be applied in future studies.

According to the results of CFA, eleven factors were identified in the extended-ISSM questionnaire. Four factors completely confirmed the original ISSM dimensions. Seven dimensions were identified and considered as influential factors on EHR success. The added factors can strengthen the ISSM framework for determining EHR success. The importance of 11 identified factors in determining the success rate in previous studies has been confirmed. For example, Lu et al. proposed a combined model of ISSM and TAM based on nurses’ points of view. The factor of “ease of use” was added to the model [32]. In the study by Hsiao et al. “top management support”, “user self-efficacy”, “perceived ease of use” factors were added to ISSM to better understand the factors affecting acceptance of hospital information systems in nurses [33]. Otieno et al. developed a questionnaire for EHR success based on nurse’s point of view that included all dimensions of ISSM [34]. The “Training” factor was included in the modified TAM in a study by Aggelidis et al. Majority of the participants in this study were nurses [35]. Our proposed framework travels beyond previous extended or modified models for nurses. The framework composed of 11 factors in an extended-ISSM. Also, we provide a questionnaire for determination of the EHR success rate with a good degree of validity and reliability.

In line with previous studies, our findings showed that the lowest EHR success rate belonged to the factor of “training”. Habibi-Koolaee et al. reported that the mean of computer skills, knowledge, and nurses' attitude towards EHR was 43.4%. They believed that holding related courses in health information systems should be considered in the nursing curriculum [36]. Poor or insufficient training courses, poor literacy and skills in technology were the frequently identified barriers to adoption and use of EHR in the study by Tsai et al. [37]. In our findings, we observed that a reverse relation between the “years of experience using computers” and “training” as well as “years of experience using EHR” and “ease of use”. Participants in the group of "over three years of experience in the computer" needed more training courses than the other groups. On the other hand, the rate of "ease of use" in participants with over six years of experience using EHR was lower than in other groups, and it might be due to the short training courses in which new EHR users participated. However, continuous training programs were not held regularly for the users. Therefore, the users after passing six years may need training. In addition, according to our results and the results of the study by Zaman et al., the variables of ICDL as general computer skills were not an effective factor in EHR success [38].

Our results showed that the mean rate of the factors of “information quality” and “service quality” were moderate. Overall, our findings are in accordance with the results of previous studies. Insufficient resource, inadequate training and technical support for users, poor literacy and skills in technology were identified barriers to EHR adoption in a scoping review [37]. Furthermore, in one study, poor quality of nursing documentation was affirmed [39].

In the current study, we found that the mean success rate of “task technology fit” and “information quality” factors was moderate based on the nurses’ perspective. There is a plausible explanation for our results. Nurses may feel that EHRs has increased their workload. However, there is no direct relationship between using the system and a nurse's duties. Furthermore, our findings are in line with findings reported by Walker et al. Their results indicated that the move from paper-based medical records to an EHR did not considerably change the amount of nurse time at the bedside and the preparation and administration of ordered medications. Nevertheless, there was an obvious trend of increased documentation time and activities following the EHR use [40]. Kossman and Susan believed that nurses preferred EHR to paper medical. They felt using EHR enhanced nursing work through increased information access, improved organization and efficiency, and alert screens. However, EHR can increase documentation time, decreased interdisciplinary communication, and impaired critical thinking. More than 70% of nurses spent at least half of their work time using EHR, and they felt EHR use enabled them to provide safer health care, but it can decrease the quality of care [41]. According to the results of a scoping review, the most negative effects of EHR implementation were related to clinical work, data and information, patient care, and economic impact [37]. Based on Jordanian nurses’ views, a lack of information technology staff and disruption to clinical care were the most common barriers [42].

Most of our nurses believed that the mean rate of the “ease of use” factor was moderate. These findings entirely tied with the results of our previous study. Plenty of usability problems were identified in the case of EHR [43]. Poor EHR usability can be associated with higher levels of stress-related to information systems that the strength of this association did not depend on user age [43]. Our results confirmed these results and an identical pattern was observed in our results. There was not a significant relationship between “age” and “ease of use” dimension. However, a positive relationship between the “ease of use” dimension and education level groups were found in our study. Unlike our finding, a stud by Khairat et al. indicated that older nurses reported higher dissatisfaction with the amount of time spent on EHR tasks related to direct patient care compared to younger nurses, and lower EHR satisfaction can impact the well-being of nurses [44]. In line with the study by Salameh et al. [45], our results revealed that the total rate of EHR success was not associated with gender. However, we observed a significant statistical relationship between “gender” and “self-efficacy”. The “self-efficacy” in females was higher than in men.

## Conclusions

In this study, a framework and an instrument to determine EHR success rates based on nurses’ perspectives were proposed in a systematic manner. The proposed framework in this study can be adopted for EHR success evaluation in future research. Moreover, this can serve as a tool for EHR comparison in various hospitals. In addition, our findings underscore the viewpoints of nurses in a developing country and provide scientific evidence on EHR success rate in such settings. Our findings also indicate that developing guidelines to improve users’ skills, strengthen information technology infrastructure, conducting information quality programs to improve documentation quality in EHR are necessary based on nurses’ point of view.

## Supplementary Information

**Additional file 1**. The list, relative frequency, and the final status of the evaluation measures.

**Additional file 2**. Extended ISSM with a 65-items questionnaire for hospital EHRs based on nurses’ point of view validated by expert panel before Confirmatory Factor Analysis (CFA).

**Additional file 3**. Extended ISSM with a 50-items questionnaire for hospital EHRs based on nurses’ point of view validated by Exploratory Factor Analysis (EFA).

## Data Availability

The generated dataset belong to the university that has a strict policy on data dissemination. The data are available from the corresponding author on reasonable request.

## Abbreviations

CI: Confidence interval
CR: Computer resources
CVI: Content validity index
CVR: Content validity ratio
EU: Ease of use
HER: Electronic health record
HOT-FIT: Human organization technology-fit
EFA: Exploratory factor analysis
IQ: Information quality
ISSM: Information systems success model
IQR: Interquartile ranges
KMO: Kaiser–Meyer–Olkin
NB: Net benefits
PCA: Principal component analysis
SE: Self-efficacy
SQ: Service quality
SS: Social support
TFF: Task technology fit
TAM2: Technology acceptance model
T: Training
TM: Top management support
UF: Usefulness
