# Supplementary material for: Development an extended-information success system model (ISSM) based on nurses’ point of view for hospital EHRs: a combined framework and questionnaire
Source: BMC Med Inform Decis Mak. 2022 Mar 22;22:71. doi: 10.1186/s12911-022-01800-1 (PMC8939199; doi:10.1186/s12911-022-01800-1)
Supplement: Supplementary file 2 — Additional file 2. Extended ISSM with a 65-items questionnaire for hospital EHRs based on nurses’ point of view validated by expert panel before Confirmatory Factor Analysis (CFA). [file 12911_2022_1800_MOESM2_ESM.docx]

| **Additional File 2:Extended ISSM with a 65-items questionnaire for hospital EHRs based on nurses’ point of view validated by expert panel before Confirmatory Factor Analysis (CFA)** | | | |
| --- | --- | --- | --- |
| **Scope** | **Evaluation Dimensions** | **Evaluation Measures** | **Questions** |
| **Technology** | **System quality** | **Sufficient resources** | CR1: Computer equipment (PC, monitor, keyword, and mouse) |
|  |  |  | CR2: Intranet (local hospital network) |
|  |  |  | CR3: Internet |
|  |  | **Reliability** | R1: The HIS you use is subject to unexpected or inconvenient downtimes which makes it hard to do your work*. |
|  |  | **Availability** | AV1: The HIS is consistently available in your work time*. |
|  |  | **System interoperability** | SIN1: The HIS is compatible with other systems you use*. |
|  |  | **Response time** | RT1: The response time of system for most operation is fast enough*. |
|  | **Information quality** | **Up-to-date** | IQ1: The hospital EHR provide up-to-date information. |
|  |  | **Sufficiently** | IQ2: The hospital EHR covers your departments’ workflow and The hospital EHR and The hospital EHR precisely offers the information and functions you need. |
|  |  | **Format** | IQ3: Information field and reports in the hospital EHR appears orderly and easy to read. |
|  |  |  | IQ4: The hospital EHR s’ field labels and fields clearly and distinctively. |
|  |  | **Locatability** | IQ5: It is easy to find the information you need in hospital EHR. |
|  |  | **Accuracy** | IQ6: The hospital EHR’ data and information is compatible with paper medical record. |
|  |  | **Right level of detail** | IQ7: The hospital EHR provides sufficient and detailed information that seems to be just exactly what you need. |
|  |  | **Authorization** | IQ8: Privileges required to access the HIS restrict accessibility to necessary patient information for daily tasks**. |
|  |  | **Timeliness** | IQ9: The availability of the output information at a time suitable for its use*. |
|  |  | **Privacy and security** | IQ10: The hospital EHR enhances the safety and confidentiality of patient data |
|  | **Service quality** | **Empathy** | SQ1: IT staff take your job problems seriously and interest to solve the problems. |
|  |  | **Responsiveness** | SQ2: IT staff provide their IT support services at the times they promise to do so. |
|  |  | **Assurance** | SQ3: You feel that IT staffs understand the health care objectives and they can communicate with you in familiar medical terms that are consistent. |
|  |  | **Responsiveness** | SQ4: The period time between a service request and IT staffs response is acceptable. (.e.g. solving a problem, giving authorized access to the hospital EHR components, and install new features) |
|  |  |  | SQ5: You received the appropriate levels of training that you need to be able to understand and use the hospital EHR. |
|  |  | **Training** | T1: The hospital EHR Manual deliver a detailed user’s manual in printed and/or electronic form. |
|  |  |  | T2: The hospital EHR has a clear instruction manual that makes it easy for you to understand and operate. |
| **Human** | **Computer knowledge and Self-efficacy** | **Self-efficacy** | SE1: If there was no one around to tell you what to do as you go. |
|  |  |  | SE2: If you could call someone for help if you got stuck. |
|  | **Positive or negative feeling about EHR** | **Computer Anxiety** | CANX1: EHR do not scare me at all*. |
|  |  |  | CANX2: Working with EHR makes me nervous*. |
|  |  | **Result Demonstrability** | RES1: I have no difficulty telling others about the results of using EHR*. |
|  |  |  | RES2: I believe I could communicate to others the consequences of using EHR*. |
|  |  | **Perceived Enjoyment** | ENJ1: I find using the EHR to be enjoyable*. |
|  |  |  | ENJ2: The actual process of using EHR is pleasant*. |
|  | **Users’ Satisfaction** | **-** | ST1: You prefer to go back to paper records (Negatively worded statements)*. |
|  |  |  | ST2: Overall satisfaction of EHR*. |
|  | **System use** | **-** | SU1: You want to use the hospital EHR. |
|  | **Voluntariness** | **-** | V1: Your use of the system is voluntary. |
|  | **Image** | - | I1: People in your hospital who use the hospital EHR have a high profile. |
|  | **Job Relevance** | - | JR1: You find hospital EHR to be useful in your job. |
| **Organization** | **Task Technology Fit (TFF)** | **Task equivocality** | TTF1: You frequently deal with business problems duo to ill-defined hospital EHR work flow |
|  |  | **Task interdependence** | TTF2: The hospital EHR problem negatively effect on your performance |
|  |  | **Compatibility and fitness with the work process** | TTF3: The hospital EHR s’ field are relevance to yours’ clinical and administrative workflow. |
|  | **Social Support (SS)** | - | SS1: your colleagues who influence my behavior think that you should use the hospital EHR. |
|  |  |  | SS2: your colleagues in your department think that you should use the system. |
|  |  |  | SS3: The senior management of this business has been helpful in the use of the system**. |
|  | **Management support** | - | TM1: Senior management ask you opinion about hospital EHR improvement. |
|  |  |  | TM2: Top management making available sufficient resources for hospital EHR development |
|  | **Environment** | - | EN1: The DOH expect you to use the EMR exchange system*. |
| **Usefulness** | **-** | - | UF1: Using the hospital EHR in your job increases your productivity. |
|  |  |  | UF2: Using the hospital EHR enhances the quality of the tasks you perform. |
|  |  |  | UF3: In your job, usage of the hospital EHR is important. |
|  |  |  | UF4: Using the hospital EHR in your job would enable you to do tasks more quickly. |
| **Ease of Use** |  | - | EU1: Interacting with the hospital EHR does not require a lot of my mental effort. |
|  |  |  | EU2: You find it easy to get the hospital EHR to do what you want it to do. |
|  |  |  | EU3: It would be easy for you to become skillful at using the hospital EHR |
|  |  |  | EU4: Interacting with the system does not require a lot of my mental effort*. |
| **Net benefit** | **Effects on outcome quality of care** | - | NB1: The hospital EHR S improves the quality of care. |
|  |  |  | NB2: By using the hospital EHR, patients have a better insight into the care provided by health care providers. |
|  |  |  | NB3: The hospital EHR reduces medical errors and improves patient safety. |
|  |  |  | NB4: The hospital EHR increases to health professionals’ ability to make patient care decisions. |
|  | **Effects on work flow and organization** | - | NB5: The hospital EHR decreases the wastefulness of resources and costs in the hospital. |
|  |  |  | NB6: The hospital EHR reduces patients waiting time for health care at the hospital. |
|  |  |  | NB7: The hospital EHR reduces the referral of patients or their families to different hospital departments. |
|  |  |  | NB8: The hospital EHR facilitates continuity of care in the next patient encounters. |
|  |  |  | NB9: The hospital EHR increases hospital administration‘s control on patient cost. |
|  |  |  | NB10: Using the hospital EHR facilitates communication between various health professionals when patient is re-admitted, is referred to other organizations and is received follow-up outpatient care. |
| **Note1:** The cells noted with a superscript “*” shows the excluded questions in EFA and superscript “**” refers to the excluded questions in CFA. | | | |
