# Supplementary material for: Development an extended-information success system model (ISSM) based on nurses’ point of view for hospital EHRs: a combined framework and questionnaire
Source: BMC Med Inform Decis Mak. 2022 Mar 22;22:71. doi: 10.1186/s12911-022-01800-1 (PMC8939199; doi:10.1186/s12911-022-01800-1)
Supplement: Supplementary file 3 — Additional file 3. Extended ISSM with a 50-items questionnaire for hospital EHRs based on nurses’ point of view validated by Exploratory Factor Analysis (EFA). [file 12911_2022_1800_MOESM3_ESM.docx]

| **Additional File 3:Extended ISSM with a 50-items questionnaire for hospital EHRs based on nurses’ point of view validated by Exploratory Factor Analysis (EFA)** | | | | | | | | |
| --- | --- | --- | --- | --- | --- | --- | --- | --- |
| Please complete the following questionnaire by placing a CROSS in the appropriate box. | | | | | | | | |
| **Technology factors** | **Computer Resources (CR)** | | How many do you have an adequate computer and network resources that are critical to use the HIS including, … | Strongly Disagree | Mostly Disagree | Slightly Agree | Mostly Agree | Strongly Agree |
|  |  |  | CR1: Computer equipment (PC, monitor, keyword, and mouse) | 1 | 2 | 3 | 4 | 5 |
|  |  |  | CR2: Intranet (local hospital network) | 1 | 2 | 3 | 4 | 5 |
|  |  |  | CR3: Internet | 1 | 2 | 3 | 4 | 5 |
|  | **Information Quality (IQ)** | | Indicate the extent to which you agree or disagree with the following statements: In your opinion, | Strongly Disagree | Mostly Disagree | Slightly Agree | Mostly Agree | Strongly Agree |
|  |  |  | IQ1: The hospital EHR provide up-to-date information. | 1 | 2 | 3 | 4 | 5 |
|  |  |  | IQ2: The hospital EHR covers your departments’ workflow and The hospital EHR and The hospital EHR precisely offers the information and functions you need. | 1 | 2 | 3 | 4 | 5 |
|  |  |  | IQ3: Information field and reports in the hospital EHR appears orderly and easy to read. | 1 | 2 | 3 | 4 | 5 |
|  |  |  | IQ4: The hospital EHR s’ field labels and fields clearly and distinctively. | 1 | 2 | 3 | 4 | 5 |
|  |  |  | IQ5: It is easy to find the information you need in hospital EHR. | 1 | 2 | 3 | 4 | 5 |
|  |  |  | IQ6: The hospital EHR’ data and information is compatible with paper medical record. | 1 | 2 | 3 | 4 | 5 |
|  |  |  | IQ7: The hospital EHR provides sufficient and detailed information that seems to be just exactly what you need. | 1 | 2 | 3 | 4 | 5 |
|  |  |  | IQ8: Privileges required to access the HIS restrict accessibility to necessary patient information for daily tasks**. | 1 | 2 | 3 | 4 | 5 |
|  | **Service Quality (SQ)** | | To what extent do you agree or disagree with the following statements? In your experience, | Strongly Disagree | Mostly Disagree | Slightly Agree | Mostly Agree | Strongly Agree |
|  |  |  | SQ1: IT staff take your job problems seriously and interest to solve the problems. | 1 | 2 | 3 | 4 | 5 |
|  |  |  | SQ2: IT staff provide their IT support services at the times they promise to do so. | 1 | 2 | 3 | 4 | 5 |
|  |  |  | SQ3: You feel that IT staffs understand the health care objectives and they can communicate with you in familiar medical terms that are consistent. | 1 | 2 | 3 | 4 | 5 |
|  |  |  | SQ4: The period time between a service request and IT staffs response is acceptable. (.e.g. solving a problem, giving authorized access to the hospital EHR components, and install new features) | 1 | 2 | 3 | 4 | 5 |
|  |  |  | SQ5: You received the appropriate levels of training that you need to be able to understand and use the hospital EHR. | 1 | 2 | 3 | 4 | 5 |
|  | **Training (T)** | | To what extent do you agree or disagree with the following statements? In your opinion, | Strongly Disagree | Mostly Disagree | Slightly Agree | Mostly Agree | Strongly Agree |
|  |  |  | T1: The hospital EHR Manual deliver a detailed user’s manual in printed and/or electronic form. | 1 | 2 | 3 | 4 | 5 |
|  |  |  | T2: The hospital EHR has a clear instruction manual that makes it easy for you to understand and operate. | 1 | 2 | 3 | 4 | 5 |
| **Organization factors** | **Task Technology Fit (TFF)** | | To what extent do you agree or disagree with the following statements? In your opinion, | Strongly Disagree | Mostly Disagree | Slightly Agree | Mostly Agree | Strongly Agree |
|  |  |  | TTF1: You frequently deal with business problems duo to ill-defined hospital EHR work flow | 1 | 2 | 3 | 4 | 5 |
|  |  |  | TTF2: The hospital EHR problem negatively effect on your performance | 1 | 2 | 3 | 4 | 5 |
|  |  |  | TTF3: The hospital EHR s’ field are relevance to yours’ clinical and administrative workflow. | 1 | 2 | 3 | 4 | 5 |
|  | **Social Support (SS)** | | SS1: your colleagues who influence my behavior think that you should use the hospital EHR. | 1 | 2 | 3 | 4 | 5 |
|  |  |  | SS2: your colleagues in your department think that you should use the system. | 1 | 2 | 3 | 4 | 5 |
|  |  |  | SS3: The senior management of this business has been helpful in the use of the system (this question was excluded from the final version of questionnaire) *. | 1 | 2 | 3 | 4 | 5 |
|  | **Top Management Support (TM)** | | TM1: Senior management ask you opinion about HIS improvement. | 1 | 2 | 3 | 4 | 5 |
|  |  |  | TM2: Top management making available sufficient resources for HIS development | 1 | 2 | 3 | 4 | 5 |
| **Human factor** | **Self-Efficacy (SE)** | | You could complete a task using the HIS, | 1 | 2 | 3 | 4 | 5 |
|  |  |  | SE1: If there was no one around to tell you what to do as you go. | 1 | 2 | 3 | 4 | 5 |
|  |  |  | SE2: If you could call someone for help if you got stuck. | 1 | 2 | 3 | 4 | 5 |
| **Ease of use (EU)** | | | Indicate the extent to which you agree or disagree with the following statements:  In your opinion, | Strongly Disagree | Mostly Disagree | Slightly Agree | Mostly Agree | Strongly Agree |
|  |  |  | EU1: Interacting with the hospital EHR does not require a lot of my mental effort. | 1 | 2 | 3 | 4 | 5 |
|  |  |  | EU2: You find it easy to get the hospital EHR to do what you want it to do. | 1 | 2 | 3 | 4 | 5 |
|  |  |  | EU3: It would be easy for you to become skillful at using the hospital EHR | 1 | 2 | 3 | 4 | 5 |
| **Usefulness (UF)** | | **Performance expectancy** | Indicate the extent to which you agree or disagree with the following statements:  In your opinion, | Strongly Disagree | Mostly Disagree | Slightly Agree | Mostly Agree | Strongly Agree |
|  |  |  | UF1: Using the hospital EHR in your job increases your productivity. | 1 | 2 | 3 | 4 | 5 |
|  |  |  | UF2: Using the hospital EHR enhances the quality of the tasks you perform. | 1 | 2 | 3 | 4 | 5 |
|  |  |  | UF3: In your job, usage of the hospital EHR is important. | 1 | 2 | 3 | 4 | 5 |
|  |  |  | UF4: Using the hospital EHR in your job would enable you to do tasks more quickly. | 1 | 2 | 3 | 4 | 5 |
|  |  | **System use** | UF5: You want to use the hospital EHR. | 1 | 2 | 3 | 4 | 5 |
|  |  | **Voluntariness** | UF6: Your use of the system is voluntary. | 1 | 2 | 3 | 4 | 5 |
|  |  | **Image** | UF7: People in your hospital who use the hospital EHR have a high profile. | 1 | 2 | 3 | 4 | 5 |
|  |  | **Job Relevance** | UF8: You find hospital EHR to be useful in your job. | 1 | 2 | 3 | 4 | 5 |
| **Net benefits (NB)** | | **Effects on outcome quality of car** | In your opinion, | Strongly Disagree | Mostly Disagree | Slightly Agree | Mostly Agree | Strongly Agree |
|  |  |  | NB1: The hospital EHR S improves the quality of care. | 1 | 2 | 3 | 4 | 5 |
|  |  |  | NB2: By using the hospital EHR, patients have a better insight into the care provided by health care providers. | 1 | 2 | 3 | 4 | 5 |
|  |  |  | NB3: The hospital EHR reduces medical errors and improves patient safety. | 1 | 2 | 3 | 4 | 5 |
|  |  |  | NB4: The hospital EHR increases to health professionals’ ability to make patient care decisions. | 1 | 2 | 3 | 4 | 5 |
|  |  |  | NB5: The hospital EHR decreases the wastefulness of resources and costs in the hospital. | 1 | 2 | 3 | 4 | 5 |
|  |  | **Effects on work flow and organization** | NB6: The hospital EHR reduces patients waiting time for health care at the hospital. | 1 | 2 | 3 | 4 | 5 |
|  |  |  | NB7: The hospital EHR reduces the referral of patients or their families to different hospital departments. | 1 | 2 | 3 | 4 | 5 |
|  |  |  | NB8: The hospital EHR facilitates continuity of care in the next patient encounters. | 1 | 2 | 3 | 4 | 5 |
|  |  |  | NB9: The hospital EHR increases hospital administration‘s control on patient cost. | 1 | 2 | 3 | 4 | 5 |
|  |  |  | NB10: Using the hospital EHR facilitates communication between various health professionals when patient is re-admitted, is referred to other organizations and is received follow-up outpatient care. | 1 | 2 | 3 | 4 | 5 |
|  |  | **Privacy and security** | NB11: The hospital EHR enhances the safety and confidentiality of patient data. | 1 | 2 | 3 | 4 | 5 |
| **Note2:** The cells noted with a superscript “*” shows the excluded questions in CFA. | | | | | | | | |
